# *Campomanesia adamantium* Peel Extract in Antidiarrheal Activity: The Ability of Inhibition of Heat-Stable Enterotoxin by Polyphenols

**DOI:** 10.1371/journal.pone.0165208

**Published:** 2016-10-20

**Authors:** Caroline Honaiser Lescano, Ivan Pires de Oliveira, Tiago Zaminelli, Débora da Silva Baldivia, Luan Ramos da Silva, Mauro Napolitano, Camila Bitencourt Mendes Silvério, Nilton Lincopan, Eliana Janet Sanjinez-Argandoña

**Affiliations:** 1 Department of Pharmacology, University of Campinas, Campinas, São Paulo, Brazil; 2 Institute of Chemistry, University of Campinas, Campinas, São Paulo, Brazil; 3 Faculty of Biological and Environmental Sciences, Federal University of Grande Dourados, Dourados, Mato Grosso do Sul, Brazil; 4 Faculty of Engineering, Federal University of Grande Dourados, Dourados, Mato Grosso do Sul, Brazil; 5 Institute of Biomedical Sciences, University of São Paulo, São Paulo, São Paulo, Brazil; Duke University School of Medicine, UNITED STATES

## Abstract

*Campomanesia adamantium* (Myrtaceae) is a medicinal plant distributed in Brazilian Cerrado. Different parts of this plant are used in popular medicine for treatment of several diseases like fever, diarrhea, hypercholesterolemia and rheumatism. The aim of this work was to evaluate the inhibition of heat-stable enterotoxin type A (STa) by gallic acid present in the peel of *C*. *adamantium* fruit and assays to assess the antidiarrheal activity, anti-inflammatory and cytotoxic properties of peel extract using the T84 cell line model. The possible inhibition exerted by the gallic acid of the peel extract on the STa peptide was inferred by molecular dynamics simulations. The antidiarrheal effects were investigated measuring cGMP accumulation in cells after stimulation by STa toxin and antibacterial activity was assessed. The anti-inflammatory activity was assessed by inhibition of COX-1 and COX-2. MTT and LDH assays were used to evaluate any possible cytotoxic action while the CyQUANT test was used to investigate the effect on cell proliferation. A representation showing how the possible interactions between STa and the gallic acid of the extract might reduce the action of the enterotoxin is presented. *C*. *adamantium* peel extract significantly decreased the levels of cGMP in T84 cells. However, no effect on the species of microorganisms was observed. The extract also inhibited COX-1 (IC_50_ 255.70 ± 0.04 ng/mL) and COX-2 (IC_50_ 569.50 ± 0.11 ng/mL) enzymes. Cytotoxicity assay have shown significant changes in cells treated with the extract, which inhibited the cell proliferation until 72 hours of treatment. Direct interactions of phenolic compounds present in the extract with the STa toxin may limit its activity. Curative effect in the diarrhea treatment and its anti-inflammatory action is based on the pharmacological properties, mechanism of action of the *C*. *adamantium* peel extract, and no toxic effects of the peel extract presented on this work.

## Introduction

World Health Organization estimates that more than 80% of the world population use medicinal plants for their health care, especially in developing countries. [1,2]. In Brazil, many plants have been associated with a potential anti-diarrheal effect [2–5], including the *Campomanesia adamantium*, a species belonging to the Myrtaceae family, and popularly known as guavira, guabiroba, gabiroba or guariroba [6].

Diarrhea is a major health issue, especially in developing countries where it is estimated to be the second major cause of death in children under five years old, killing more children than AIDS, malaria and measles combined [2,7–9]. It can be caused by different pathogens including bacteria, virus and protozoa [10].

Almost 60% of deaths associated with diarrhea are linked to enterotoxigenic *Escherichia coli* (ETC), which acts by releasing two plasmid-encoded enterotoxins: heat-labile (LT) and heat-stable enterotoxin (ST) [11]. Two families of ST enterotoxins, STa and STb, have been identified, which have distinct mechanism of action [12]. STa binds to the membrane receptor of guanylate cyclase type C (GC-C) in intestinal epithelial cells, stimulating the synthesis of 3'-5' cyclic guanosine monophosphate (cGMP) from guanosine triphosphate (GTP), thus starting diarrheal framework [13–16].

Several works have already shown that members of the *Campomanesia* genus may exert different biological activities [17–20]. For example *Campomanesia xanthocarpa* showed efficacy in weight loss and blood glucose levels decrease in rats, as well as having antiplatelet and antithrombotic properties [18,21]. On the other hand it was demonstrated the antimicrobial activity of essential oil from different plant parts of *Campomanesia pubescens* (root, stem, leaf and fruit) [22], while the *Campomanesia adamantium* leaves showed anti-inflammatory effects [19]. However, there are no reports in the literature corroborating the effectiveness of this species for the treatment of diarrhea.

The aim of this study was the evaluation of antidiarrheal potential of *Campomanesia adamantium in vitro*, as well as its antimicrobial, anti-inflammatory, cytotoxic potential. We propose a mechanism of action consisting in the inhibition of STa by the phenolic compounds present in the peel of fruits.

## Materials and Methods

### Chemicals

MTT (3-(4,5-dimetylthiazol-2-yl)-2,5-diphenyltetrazolium bromide), DMSO (dimethyl sulfoxide), Dulbecco’s modified eagle medium (DMEM-F12), gallic acid, quercetin, diclofenac sodium salt, penicillin and amoxicillin were purchased from Sigma-Aldrich^®^ (MO, USA). Fetal bovine serum (FBS) was purchased from Invitrogen (Gibco^®^, NY, USA) and Heat-stable entrotoxin (STa) from Bachem. Biochemical assay kits were from Cayman Chemical cGMP, LDH cytotoxicity, COX inhibitor screening and Molecular Probes (CyQUANT^®^ direct cell proliferation assay). All reagents used were analytical grade.

### Plant material

*Campomanesia adamantium* fruits were collected in regions of Cerrado biome located in the State of Mato Grosso do Sul, Brazil (22° 4′ 34.824″ S and 55° 8′ 33.936″ W), from Medicinal Plants Garden of Federal University of Grande Dourados (UFGD). A voucher specimen was deposited in the UFGD (n. 47620). The fruits were selected to obtain a uniform batch regarding size and absence of injuries, washed, and sanitized with a solution of 0.66% sodium dichloroisocyanurate dihydrate. Samples were pulped manually; pulp and peel were stored at -5°C until processing.

### Extract preparation

Peels were previously dehydrated at 40°C in a tray dryer (NG Scientific) with an air flow of 0.5 ms^-1^ for 72 hours and triturated to a fine powder. Fresh fruit and peels were extracted with methanol 100% for 21 days and filtrated. Extracts were mixed, filtered, and concentrated under vacuum and lyophilized. The final powder was diluted in vehicle according to the experiment and then adjusted to the desired concentration to perform the tests.

### Phenolic compounds and flavonoids

Concentration of phenolic compounds in the peel extract was determined according to Folin-Ciocalteu colorimetric method described by Singleton and coworkers [23]. Briefly, 0.5 mL of the peel extract (10 mg/mL) was mixed with 2.5 mL of Folin-Ciocalteu reagent and 2 mL of sodium carbonate (Na_2_CO_3_) 14% (w/v). After 2 hours of incubation at room temperature in the dark, the absorbance at 760 nm was measured. Gallic acid (0.4 to 22 μg/mL) was used as a standard to construct a calibration curve and the average of 3 readings was used to determine the content of phenolic compounds, which was expressed in mg of gallic acid equivalent (GEA) per g of peel extract. The concentration of total flavonoids in the extracts was determined according to the method described by Chang et al (2002) [24], 0.5 mL of the extract (10 mg/mL) was mixed with 0.1 mL of aluminum chloride 10% (w/v), 0.1 mL of sodium acetate 1 M and 2.8 mL of distilled water. The absorbance was read at 415 nm after incubation of 40 min at room temperature under darkness conditions. Quercetin (0.4 to 22 μg/mL) was used as a standard to construct a calibration curve and the average of three readings was used to determine the content of flavonoids compounds, which was expressed in mg of quercetin equivalent (QE) per g of extract.

### cGMP assay

Human colorectal carcinoma cell, or T84 cell, was from American Type Collection (CCL248) and were grow in Dulbecco´s Modified Eagle´s Medium/Nutrient Mixture F-12 (DPBS) Ham supplemented with 10% heat-inactivated fetal calf serum and 100 units/mL penicillin, 100 μg/mL streptomycin in a humidified atmosphere of 95% air, 5% CO_2_ at 37°C. The line of intestinal T84 cells were grown to confluence in 12-well plates and washed three times with DPBS. Cells were treated with vehicle (DPBS), extract (0.25, 0.5, 1, 2.5, 5 and 10 mg/mL), tadalafil (50 μM), gallic acid (10 μM) or 5-[3,5-bis(Trifluoromethyl)phenyl]-1,3-dimethyl-5,11-dihydro-1H-indeno-[2’,1’:5,6] pyrido[2,3-d]pyrimidine-2,3,6-trione (FPIPP) [25], for 10 min and then cGMP accumulation was stimulated with *E*. *coli* enterotoxin (STa, 1 μM) for 10 min. Medium was aspirated and cGMP was extracted with HCl 0.1 M by 30 min and centrifuged. cGMP was measured by ELISA. Proteins were extracted with NaOH 0.1 M and its concentrations measured by Bradford method [26]. Results are expressed in cGMP pmol/10 min/mg.

### Molecular Dynamic simulations

The molecular dynamics simulation (MD) was performed based on crystallographic structure of the *Escherichia Coli* Heat-Stable Enterotoxin obtained from Protein Data Bank (PDB:1ETN) determined by X-Ray Diffraction at a resolution of 0.89 Å. The initial system of simulation was built with Packmol [27], containing the Heat-Stable Enterotoxin peptide, gallic acid (100 molecules), water (1500 molecules), Na^+^ and Cl^-^ ions to neutralize the charge of simulation box (~40 Å of side) and density approximately 1.0 g/mL. Gallic acid is a molecule model to study the peptide-polyphenol interactions. The MD was performed in the NPT ensemble at 1 atm and 298.15 K (25°C). The CHARMM force field was used [28] and TIP3 model for water molecules [29]. The force field parameters for gallic acid were performed by CGenFF program [30]. 100 ns of simulation were computed with NAMD [31] and the visualizations with VMD [32] programs. The solute-solvent distribution functions (g_ss_) were calculated with the MDAnalysis package (Martínez, 2014), considering the shortest distance between solute (STa) and solvent (gallic acid).

### Antibacterial assay

The antibacterial activity was assessed against *E*. *coli* ATCC 7789, *Salmonella typhimurium* ATCC 14028 and *Staphylococcus aureus* ATCC 6536. The test was carried out by disc diffusion method on Mueller Hinton agar in accordance with Clinical Laboratory Standards Institute guidelines (Clinical and Laboratory Standard Institute. Performance Standards for Antimicrobial Disk Susceptibility Tests: Vol. 32, No. 1, M02 A11. Pennsylvania, USA: Clinical and Laboratory Standard Institute, 2012). Briefly, fresh colonies were first suspended in liquid LB medium to achieve an OD600 ≈ 0.1 (roughly equivalent to a turbidity of 0.5 MacFarland standards) and then streaked on the surface of Mueller Hinton (MH) plates by a sterile swap. Subsequently sterile disks soaked with 20 μL of DMSO dissolved *C*. *adamantium* peel extract at different concentrations (50, 25, 12.5, 6.25, 3.12 mg/mL) were placed into the plates. Discs containing 30 μg chloramphenicol or 1 μg oxacillim were used as positive controls. Bacteria were allowed to grow 16–18 hours at 37°C.

### Cell metabolic activity

MTT assay was performed according to Mosmann [33]. T84 Cells were incubated with peel extract (0.25, 0.5, 1, 2.5, 5 and 10 mg/mL) for 1, 24 and 48 hours. After the incubation period, cells were washed and incubated with 5 mg/mL of MTT solution for 2h in a CO_2_ incubator. Then MTT dye was removed and 100 μL of solubilization solution (acidified isopropanol) were added to the wells. Plates were analyzed by measuring the absorbance at 570 nm.

### LDH assay

T84 cells were grown in a 96-well plate and incubated with extract (10 mg/mL) for 1 and 24 hours. Next, plates were centrifuged at 400 x g for five minutes and 100 μL of cell supernatant was transferred to a new 96-well assay plate where 100 μL of reaction solution was added to each well. Plates were incubated with gentle shaking on an orbital shaker for 30 min at room temperature and the absorbance read at 490 nm.

### Cell proliferation

The cell proliferation was performed by CyQUANT assay (which green fluorescence when bound to nucleic acids) in cell of human colon carcinoma (T84). T84 cells were grown (48 h) in a 96-well plate and incubated with extract (10 mg/mL), STa (1 μM), gallic acid (10 μM) and vehicle (DPBS) for 24, 48 and 72 hours. Next, proliferation assay was conducted according to manufacturer instructions and plates incubated during 60 minutes at 37°C in CO_2_ incubator. Fluorescence was measured using excitation at 480 nm and emission at 535 nm.

### Cox inhibition

Inhibition of COX activity was assayed by using the colorimetric COX inhibitor Screening Assay kit according to the manufacturer instructions. The extract (0.001–10 mg/mL), gallic acid (0.0001–100 μM) and diclofenac (0.0003–30 μM) was incubated for 10 min and the absorbance was read using a plate reader at 412 nm. Diclofenac was used as the reference compound, and all compounds were diluted in a buffer kit. The IC_50_ values were calculated from the concentration–inhibition response curves.

### Statistical analysis

Data are shown as mean ± SEM and statistical significance was calculated using ANOVA followed by Tukey’s test assuming P<0.05 as significant, calculated with GraphPad Prism (Version 6.0—GraphPad Software, San Diego, CA).

## Results

### Total phenols and flavonoids

Analysis of phenols has shown that phenolic compounds are present in peel extracts of *Campomanesia adamantium* (Table 1). The total phenolic content were 1.35 ± 0.15 mg of gallic acid equivalent (GAE) per g of peel extract while the flavonoids content was 0.96 ± 0.01 mg of quercetin equivalent (QE) per g of extract.

**Table 1:** Total phenolic and flavonoid content of *Campomanesia adamantium* peel extract.

Phenol analyses	Quantified
Total phenolic (mg GAE/g)	1.35 ± 0.15
Total flavonoids (mg QE/g)	0.96 ± 0.01

Results are presented as mean ± SEM. n = 3

### cGMP levels

**Fig 1:**
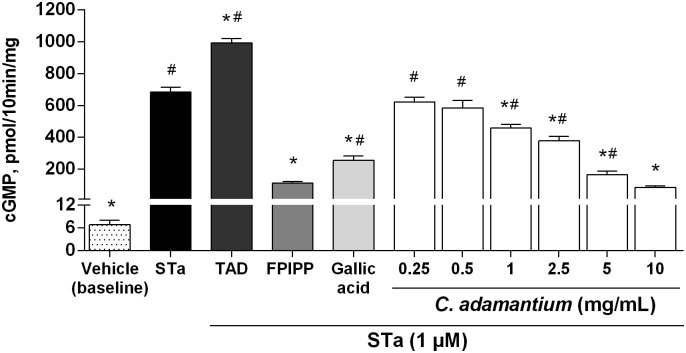
Effect of *Campomanesia adamantium* peel extract on cGMP levels in T84 cells. Cells were pretreated with extract (0.25, 0.5, 1, 2.5, 5 and 10 mg/mL) or vehicle (DPBS) for 10 min and then treated with 1 μM of STa for 10 min. STa was added in groups of *C*. *adamantium* (all concentrations), Tadalafil e FPIPP. TAD (tadalafil) and FPIPP (antidiarrheal drug) was used as control drugs that increase and decrease cGMP levels, respectively. Results are presented as mean + SEM. n = 4–6; One-way ANOVA followed by Tukey´s test; #P <0.05 compared to baseline group; *P <0.05 compared to STa group.

To evaluate any possible interference of *C*. *adamantium* peel extract on the action of STa toxin, the accumulation of cGMP in T84 cells was measured after stimulation of particulate guanylate cyclase by toxin STa. It was found that *C*. *adamantium* peel extract contrasted the action of STa toxin in T84 cells at concentration of 10 mg/mL. Fig 1 shows that peel extract significantly reduced cGMP accumulation induced by STa toxin in T84 cells (88% of inhibition, 85.39 ± 9.98 pmol/10 min/mg of protein). The effect of gallic acid on the inhibition of STa action was evaluated, and gallic acid (10 μM) significantly inhibited the accumulation of cGMP in T84 cells (63% of inhibition, 255.08 ± 27.55 pmol/10 min/mg of protein).

*C*. *adamantium* peel extract without stimulation of STa, has shown that the intern level of GMP was not significantly affected (P<0.05), presenting values of 10.82 ± 0.86 pmol/10 min/mg of protein, and baselines of cGMP 6.80 ± 1.20 pmol/10 min/mg of protein.

### Molecular dynamic simulations

**Fig 2:**
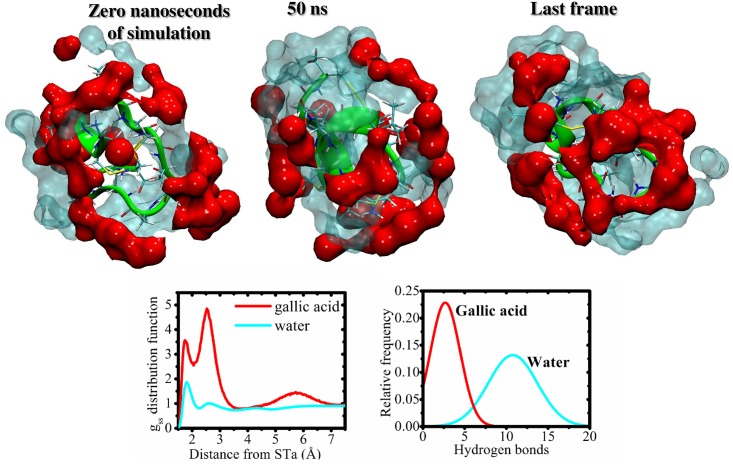
Solute-solvent distribution function (gss) for all residues and water (cyan) or gallic acid (red). Molecular view at three stages of simulation, initial, 50 ns and last frame, showing the increase of phenol concentration at ~1.7 Å and ~2.5 Å for all peptide. The H-bonds were calculated for water and gallic acid with STa.

**Fig 3:**
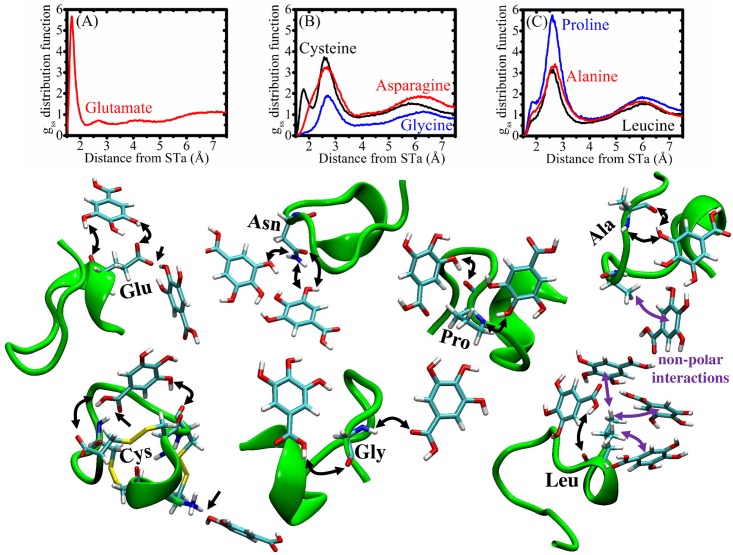
Solute-solvent distribution function (gss) for specific residues. (A) charged glutamate residue; (B) polar residues and (C) non-polar residues. Non-polar interactions with hydrophobic residues are highlighted in purple. Several interactions with peptide backbone were identified.

The interactions between gallic acid and STa peptide are demonstrated qualitatively in Fig 2. Specifically, Fig 3 shows that gallic acid-STa interactions occurs at short distances (~1.7 and 2.5 Å) and for long distances ~5.8 Å. These affinities are dependent on the type of residue.

The STa-gallic acid interactions were investigated for specific residues, shown in Fig 2. It’s possible to see that charged residue (glutamate) is very important for interactions at short distances with phenolic compounds (hydrogen bond is the main interaction as shown in Fig 3). Cysteine residue also contributed for short interactions with gallic acid. Moreover, polar compounds, such as glycine and asparagine, bind with phenolic compound at distances with maximum peaks at ~2.7 Å. In addition, the non-polar residues (proline, leucine and alanine) also interact with gallic acid at ~2.6 Å.

### Antibacterial

The possible antibiotic effect of *C*. *adamantium* peel extract was evaluated against Gram-negative enterobacteria and the Gram-positive *Staphylococcus aureus*. Following 16–18 hours of incubation at 37°C our positive controls effectively inhibited bacterial growth, however no effect on any of the species used was observed, even in presence of the highest concentration of extract used (50 mg/mL).

### Cell metabolic activity and cytotoxicity

**Fig 4:**
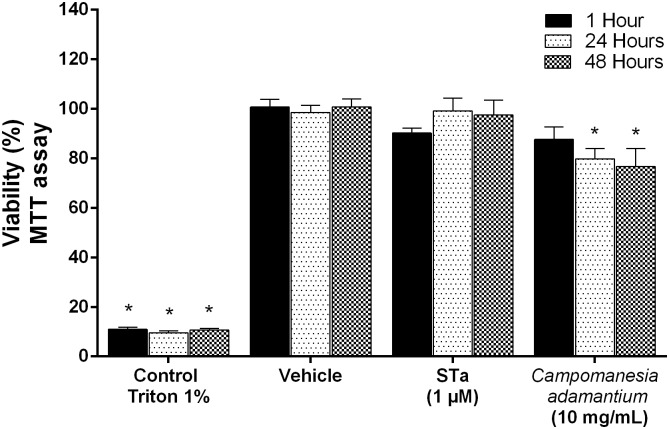
Effect of *Campomanesia adamantium* peel extract on cell metabolic activity. T84 cells were treated with 10 mg/mL of extract or vehicle (medium) for 1h, 24h and 48h. Results are presented as mean + SEM. n = 9; Two-way ANOVA followed by Tukey´s test; *P <0.05 compared to vehicle group.

To study changes in metabolism and cytotoxicity in human colon carcinoma cells, the effects of the methanol extract from the peel of *C*. *adamantium* were investigated by MTT and LDH assays. As displayed in Fig 4 the treatment with the extract at concentration of 10 mg/mL showed effects on cellular metabolism during the incubation period of 24 to 48 hours (20.22% and 23.25%, respectively).

**Fig 5:**
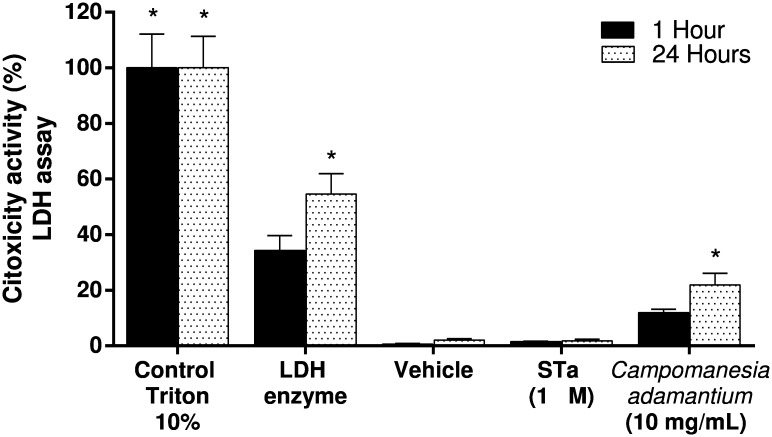
Effect of *Campomanesia adamantium* peel extract in citotoxicity. T84 cells were treated with 10 mg/mL of extract or vehicle (medium) for 1h and 24h. Results are presented as mean + SEM. n = 9; Two-way ANOVA followed by Tukey´s test; *P <0.05 compared to vehicle group.

To verify the inhibitory activity of the *C*. *adamantium* peel extract in T84 cell was not due to its cytotoxicity, the effect of the extract on cell viability was determined using a lactate dehydrogenase release assay (LDH). As shown in Fig 5, concentrations of 10 mg/mL of the extract produced cytotoxicity in T84 cells only after 24 hours of treatment.

### Cell proliferation

**Fig 6:**
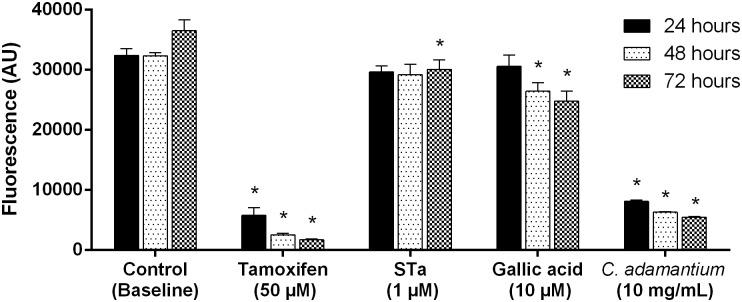
Effect of *Campomanesia adamantium* peel peel extract in T84 cell proliferation. Results are presented as mean + SEM. n = 6; Two-way ANOVA followed by Tukey´s test; *P <0.05 compared to Control (baseline) group.

The effect of *C*. *adamantium* peel extract in cancer cells proliferation (cell of human colon carcinoma) treated with the peel extract is shown in Fig 6. The results show that *C*. *adamantium* peel extract produce inhibition after 24, 48 and 72 hours of treatment of the cellular growth.

### COX-1 and COX-2 Inhibition

Data presented in Table 2 show the results of anti-inflammatory activity of the peel extract, evaluated for its ability to inhibit COX-2 enzyme by an *in vitro* colorimetric COX (ovine) inhibitor assay. Both isoforms of the COX enzyme were sensitive to inhibition induced by *C*. *adamantium* peel extract, as shown in Table 2. At the concentration of 1 mg/mL, the peel extract was able to reduce the activity of both COX-1 and COX-2 enzymes by approximately 93% and 92%, respectively.

**Table 2:** Effect of *Campomanesia adamantium* in peel extract on COX-1 and COX-2 activities.

Compounds	IC_50_	
	COX-1	COX-2
*C*. *adamantium* (ng/mL)	255.70 ± 0.04	569.50 ± 0.11
Diclofenac (nM)	0.54 ± 0.07	90.23 ± 0.48
Gallic acid (μM)	1.59 ± 0.21	3.53 ± 0.41

Results are presented as mean ± SEM. n = 3

## Discussion

The phenol analyses have shown that phenolic compounds are present in the peel extracts of *C*. *adamantium*. This results corroborates with Coutinho and collaborators, which have identified nine specifics flavonoids from *C*. *adamantium* leaves [34]. Moreover, Cardoso and collaborators investigate the flavonoids content in extracts and fractions of *C*. *adamantium* fruits [17]. Phenolic compounds have antioxidants properties and can protect the intestinal environment against oxidative stress in intestinal hypersecretion [35,36]. There is a relationship between the extracts rich in phenolic compounds (hydroxybenzoic acid, anthocyanins and flavonoids) and the maintenance of the antioxidant activity of enzymes such as superoxide dismutase (SOD), catalase (CAT) and glutathione peroxidase (GPx). In this sense, the ability of phenolic compounds to interact with proteins must be evaluated carefully. MD simulations was applied to obtain a molecular view of the interactions between a small peptide STa and phenols presents in *C*. *adamantium* peel extracts.

Our result of cyclic nucleotides levels shows that when T84 cells are incubated with *C*. *adamantium* peel extract (0.25 mg/mL up to 10 mg/mL) for 10 min, the levels of intracellular cGMP induced by STa is inhibited in a range between 12 and 88%. The decrease in cGMP levels in enterocytes is particularly important due to the role of cyclic nucleotides in promoting the diarrhea cascade. The interaction of STa (or with the endogenous mediators guanylin and uroguanylin) with the extracellular domain of Guanylate Cyclase type C (GC-C) receptor induces conformational change in the extracellular portion of the homotrimeric GC-C complex. This leads to the dimerization of two intracellular catalytic domains and the formation of two active catalytic clefts, which ultimately stimulates Cl^-^ and HCO_3_^-^ secretion and inhibition of Na^+^ absorption by enterocytes [37].

The decrease in cGMP synthesis observed in this study may be related to any of the following events: (i) competition to the binding site at GC-C receptor, (ii) desensitization/allosteric modulation of the GC-C receptor, (iii) block or interference of mediators of the signalizing cascade or (iv) modulation of PDE5 activity. Possibly, *C*. *adamantium* peel extract hampers STa binding to the receptor, perhaps competing for the same binding site, thus, attenuating the adverse effect of the toxin by partially blocking the intracellular cGMP accumulation, as observed in the present report. Although *C*. *adamantium* peel extract was able to block the accumulation of cGMP when stimulated by toxin, no changes in its basal level were observed. This is extremely important considering that small changes in cGMP levels can promote significant physiological effects due to the importance of this intracellular signaling pathway [38,39].

The hypothesis of the interaction of phenolic compounds present in the peel extract with STa peptide, was investigated by MD simulations. The gallic acid was chosen as a representative model of the polyphenols present in the peel extract of *C*. *adamantium* due to its relationship with antidiarrheal activity previously described [40] and gallic acid inhibited the accumulation of cGMP in cells. The action of STa is directly related to its interaction with guanylate cyclase (GC) [13], leading to the cGMP accumulation. Therefore, compounds that bind strongly on STa should disrupt STa-GC interactions, and this chemical property may be explain the results observed previusoly. In Fig 2 it’s possible to observe the water-STa and the gallic acid-STa interactions. The high relative density of water molecules at ~1.8 Å is due to hydrogen bonds interactions, the first layer of solvation. However, the most interesting is the gallic acid molecules interactions with STa peptide. For short distance these phenolic compounds are more concentrated, peaks at ~1.8 Å and principally ~2.5 Å performing hyrogen bonds interactions. For long distance, ~5.8 Å, there is a high concentration of gallic acid, too. These results show that the STa is solvated strongly for gallic acid and may be possible that similar phenolic compounds exhibit the same property. In this sense, its is plausible that phenolic compounds presents in *C*. *adamantium* peel extracts may be inhibiting the mechanism of STa in diarrhea process through the direct interactions (STa-phenolic compounds), and preventing the STa stimulates guanylate cyclase. In summary, we propose that phenolic compounds presents in *C*. *adamantium* extracts may be inhibiting the mechanism of STa in diarrhea process through the direct interactions (STa-phenolic compounds), and preventing the STa stimulates guanylate cyclase.

**Fig 7:**
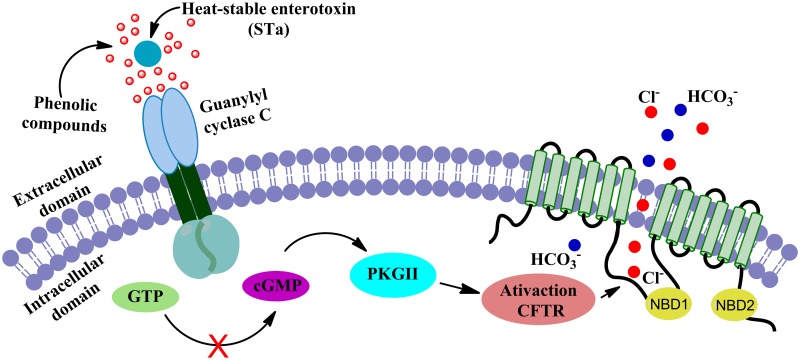
Representation of the mechanism of action of phenolic compounds presents in *C*. *adamantium* peel extracts. The STa peptide and guanylate cyclase are composed by residues of amino acids that may be binding preferentially with phenolic molecules.

The interaction between compounds rich in hydroxyls groups and residues of proteins are known in the literature [41,42], and considering the amino acid chain that makes up the STa and the GC receptor, here we propose that interactions between phenolic compounds and residues of GC protein can be occurring in the extracellular domain of guanylate cyclase. Fig 7 shows the possible mechanism of action of the phenolic compounds presents in *C*. *adamantium* peel extracts that contributes to antidiarrheal effects: a) STa peptide inhibition and b) GC extracellular domain direct interactions. This second assumption is possible because STa and the GC receptor have the same primary composition, residues of amino acids.

Bacterial infection could affect gastrointestinal motility leading to disturbances of the digestive system such as diarrhea. Agents such enterotoxigenic *Escherichia coli* causes disease by colonizing the small intestine and producing heat-labile toxin [43]. In the disk diffusion test, the extract of *C*. *adamantium* fruits failed to show activity against *E*. *coli*, *S*. *aureus* and *S*. *thyphimurium*. This result is in contrast with the antimicrobial activity attributed to *C*. *adamantium* in a previous report [44]. In this study essential oils extracted by hydrodistillation from flowering and fruit-bearing stages showed antimicrobial activity against *Staphilococcus aureus*, *Pseudomonas aureuginosa* and *Candida albicans*. A significant portion (~ 50%) of the compounds identified in these oils was represented by terpenes. The crude extract used in the present work was extracted with methanol, which is less effective than hydrodistillation in the isolation of hydrophobic molecules like terpenes. Thus it is possible that the lack of antibacterial activity observed here could be due to the low abundance of these compounds in our extract. Another study reported the anti-*Mycobacterium tuberculosis* activity of ethyl acetate and methanol extracts of *C*. *adamantium* fruits [45] suggesting that other species (different from those tested in this work) may be negatively affected by the plant extract. Moreover, other members of the genus *Campomanesia*, like *C*. *xanthocarpa*, *C*. *eugenioides* and *C*. *lineatifolia* have been reported to have antimicrobial activity over a great variety of microorganisms including bacteria and fungi [46–48].

Besides the popular use of infusion of the leaves and peel of *C*. *adamantium* fruits for anti-diarrheal treatment, popular medicine also makes use of this species for the treatment of inflammation. To demonstrate its anti-inflammatory action, we evaluated the effect of the peel extract of *C*. *adamantium* fruit in the inhibition of the enzyme cyclooxygenase (COX) *in vitro*. In the present work we show that *C*. *adamantium* peel extract has anti-inflammatory potential since it was able to reduce the activity of COX-1 and COX-2 enzymes, shown in Table 2. These isoforms have different structures, with 65% degree of homology, but their functions are different in the same cell type [49]. COX-1, known as constitutive enzyme, is present in almost all tissues and is associated with the production of prostaglandins resulting in a variety of physiological effects, whereas COX-2 is expressed in inflammation areas. Natural products that are able to inhibit the activity of these enzymes are potentially promising anti-inflammatory *in vivo*, promoting a reduction of inflammation, pain and fever. Souza et al [20] show that extract of *C*. *adamantium* fruit peels have *in vivo* anti-inflammatory activity and ascribe this result to the flavonoids (myricetin) and chalcones, main constituents identified in the methanolic fraction of the extract. Thus, corroborating our results, the inhibition of two isoforms observed in this study is related to these compounds described in the literature. Reports in the literature show that quercetin and myricetin compounds contributes to inflammatory and antinociceptive activities by modulating production of the inflammatory mediator nitric oxide (NO) and anti-inflammatory cytokine interleukin-10 (IL-10) [50].

The antiinflammatory effect of many plant species may have a fundamental role in colorectal carcinogenesis, in particular through modulation of COX-2 which contribute to the cytotoxic effect. However, our results show that treatment with *C*. *adamantium* peel extract was non-toxic in T84 cells 1 hour incubation period. In contrast cell metabolism was significantly altered. As cell viability decreased in a time-dependent fasshion, an explanation of the results observed on cell viability can be related to the high quantity of phenolic compounds. Previously, it was reported that catecholamines inhibit the growth of HT29 colon carcinoma cells [51,52]. Moreover, it has been shown that the gallic acid induces apoptosis in cells of esophageal cancer [53,54]. Likewise, it may be possible that the combination of phytochemical compounds are acting in synergism with T84 colon carcinoma cells.
